# Non‐Surgical Submarginal Instrumentation of Peri‐Implant Mucositis With Delivery of Sodium Hypochlorite/Amino Acids and Cross‐Linked Hyaluronic Acid: A Randomized Clinical Trial

**DOI:** 10.1111/clr.70109

**Published:** 2026-02-27

**Authors:** Vincenzo Iorio‐Siciliano, Andrea Blasi, Leopoldo Mauriello, Peter Windisch, Giovanni E. Salvi, Anton Sculean, Luca Ramaglia

**Affiliations:** ^1^ Department of Periodontology University of Naples Federico II Naples Italy; ^2^ Department of Periodontology Semmelweis University Budapest Hungary; ^3^ Department of Periodontology, School of Dental Medicine University of Bern Bern Switzerland

**Keywords:** antiseptics, biofilm, hyaluronic acid, inflammation, mucositis, sodium hypochlorite

## Abstract

**Objectives:**

To evaluate the effects of submarginal instrumentation (SI) with or without adjunctive delivery of sodium hypochlorite (NaOCl)/amino acids and cross‐linked hyaluronic acid (xHyA) gel in the treatment of peri‐implant mucositis (PM).

**Material and Methods:**

Forty implants supporting single‐unit crowns diagnosed with PM in 40 patients were randomly assigned to test (SI + NaOCl/amino acids and xHyA) or control group (SI alone). The primary outcome was mean BoP change. Full‐Mouth Plaque Score (FMPS), Full‐Mouth Bleeding Score (FMBS), modified plaque index (mPlI), and probing depth (PD) were assessed as secondary outcomes. Clinical parameters were assessed at baseline, 3 and 6 months. Disease resolution was also recorded.

**Results:**

Two patients were lost during follow‐up while 38 patients completed the study without adverse effects. After 6 months, all clinical parameters improved statistically significantly in both groups (*p* < 0.05). The change in mean BoP at 1, 3, and 6 months was 72.2% ± 24.3%, 70.4% ± 24.3%, and 63.0% ± 24.6% for test group and 70.0% ± 19.9%, 66.7% ± 24.8%, and 56.7% ± 30.8% for control group. The mean BoP change in experimental procedure was statistically significant at all investigation time points (*p* < 0.05). Regarding disease resolution, implants with initial PD ≤ 4 mm did not show differences among groups (*p* > 0.05); conversely, an initial PD = 5 mm yielded a statistically significant difference (*p* < 0.05). Disease resolution correlation with test group was statistically significant with a 3.77 odds ratio.

**Conclusion:**

Within the limitations of the present study, adjunctive delivery of NaOCl/amino acids and xHyA to SI yielded superior clinical outcomes compared with SI alone in the treatment of PM.

**Trial Registration:**

ClinicalTrials.gov: NCT05926297

## 1 Introduction

Clinical and histological studies (Berglundh et al. 2024; Heitz‐Mayfield 2024; Carcuac et al. 2013; Pontoriero et al. 1994) provided evidence that biofilm accumulation surrounding dental implants induces peri‐implant tissue inflammation in response to microbial insult (e.g., peri‐implant mucositis and peri‐implantitis).

Peri‐implant mucositis (PM) is an inflammatory lesion caused by biofilm involving the soft tissues around implants without bone loss (Heitz‐Mayfield and Salvi 2018). The lack of early treatment of PM may determine an involvement of peri‐implant bone (i.e., peri‐implantitis) with high probability of implant loss if left untreated (Berglundh et al. 2018). The onset of peri‐implantitis in relation to function of time was reported to start already after 3 years of function (Derks and Tomasi 2015; Derks et al. 2016). The incidence of peri‐implantitis in patients with untreated PM was 43.9% after 5 years observation period with a proportion of implant loss of 1.6% (Costa et al. 2012). Hence, PM is considered the precursor of peri‐implantitis (Salvi et al. 2019; Schwarz et al. 2018) and an effective treatment is a key element to prevent the progression of PM to peri‐implantitis (Jepsen et al. 2015). Studies on experimental PM in humans indicated that reversibility may be incomplete after 3 weeks (Salvi et al. 2012) and requires an additional 2–3 weeks of healing (Chan et al. 2019). Professional submarginal instrumentation (SI) using hand or powered instruments without local adjunctive agents and self‐administered biofilm removal (Herrera et al. 2023) determines an improvement in clinical parameters (i.e., BoP) (Verket et al. 2023). Nevertheless, resolution of PM ranged from 40% to 43% (Maximo et al. 2009; Iorio‐Siciliano et al. 2023). To improve clinical outcomes in the management of PM, many authors proposed the use of professionally administered chemical agents (i.e., antiseptics) as an adjunct to SI (Sahrmann et al. 2019). The rationale for the adjunctive use of local antiseptics (i.e., chlorhexidine gel or chips) in treatment of PM relates to its efficacy against a broad spectrum of antimicrobial activity based on microbiological and in vitro findings (Jones 1997). Likewise, sodium hypochlorite 0.95% (NaOCl) with amino acids gel reduces the biofilm vitality due to antimicrobial activities, especially against Gram‐negative bacteria (Jurczyk et al. 2016). NaOCl 0.95% combined with amino acids (i.e., glutamic acid, leucine, lysine) determines the formation of chloramines which show the capacity to penetrate and to soften the biofilm facilitating its removal (Schmidlin et al. 2017; Iorio‐Siciliano et al. 2021).

Therefore, these results indicate that the use of local antiseptics could play a pivotal role in the treatment of PM. On the contrary, clinical data of a systematic review reported that the adjunctive use of locally administered antiseptics (e.g., chlorhexidine gel/solution or NaOCl 0.95%) following SI does not seem to additionally improve the clinical outcomes (i.e., BoP change and disease resolution) when compared to SI alone (Dommisch et al. 2023). These findings are corroborated by a recent study on the effects of non‐surgical mechanical treatment of PM with adjunctive application of chlorhexidine solutions. Despite a significant improvement in BoP reduction was found, a complete disease resolution was observed in only 43% of the treated implants (Isola et al. 2025). In the last years, emerging evidence indicates that the use of hyaluronic acid (HA) provided clinical benefits in periodontal therapy (Eliezer et al. 2019). HA is synthetized by HA synthase enzyme present in many cells including fibroblast and keratinocytes of periodontal tissues (Fraser et al. 1997; Ijuin et al. 2001). Moreover, HA stimulates cell proliferation (Olczyk et al. 2008), induces angiogenesis (Deed et al. 1997) and osteogenesis (Bezerra et al. 2012) playing a crucial role in blood clot formation and in each phase of wound healing (Scully et al. 1995). Furthermore, HA inhibits the growth of bacterial stains and prevents recolonization due to the bacteriostatic effect on *Treponema denticola* , *Comphylobacter rectus*, *Prevotella intermedia* , and *Porphyromonas gingivalis* (Pirnazar et al. 1999; Eick et al. 2013). A new formulation of cross‐linked hyaluronic acid gel of non‐animal origin with high molecular weight (xHyA) was tested in preclinical studies indicating that xHyA accelerated the phase of wound healing by stimulating cell migration and proliferation (Mueller et al. 2017). These preclinical observations were clinically verified in non‐surgical treatment of periodontal intrabony defects with an improvement of clinical outcomes in the first 3 months following therapy with respect to non‐surgical therapy alone (Iorio‐Siciliano et al. 2025). Based on the properties of NaOCl 0.95%/amino acids and xHyA, several authors proposed to treat periodontal pockets using the combination of both agents in non‐surgical treatment of periodontitis (Ramanauskaite et al. 2023). Since clinical relevance was observed in periodontal therapy, it is possible to hypothesize that the local adjunct of NaOCl 0.95%/amino acids and xHyA to SI provides better results with respect to SI alone in the treatment of PM.

As of today, however, data on potential clinical benefits of local application of xHyA in conjunction with SI in the treatment of PM are limited.

Hence, the aim of this study was to verify the effects of submarginal instrumentation (SI) with or without adjunctive delivery of sodium hypochlorite (NaOCl)/amino acids and cross‐linked hyaluronic acid (xHyA) gel in the treatment of peri‐implant mucositis (PM).

## 2 Materials and Methods

### 2.1 Study Design

A superiority parallel‐arm, double‐blind randomized controlled trial was designed. Patients rehabilitated with only one implant affected by PM were randomly allocated to the test or control group. Implants of the test group received SI with adjunctive application of NaOCl 0.95%/amino acids and xHyA, whereas control implants were treated by means of SI alone. The null hypothesis of no statistically significant differences with respect to BoP change between test and control procedure was tested. The study is reported according to the CONSORT statement, and it was conducted in observance of the Principles of the Declaration of Helsinki on experimentation involving human subjects. Prior to receiving the test or control procedure, written consent was obtained from all patients. The study protocol was submitted to and approved by the ethical committee of the University of Naples Federico II (approval number: 110/2023) and it was registered at ClinicalTrial.gov (ID NCT05926297) https://clinicaltrials.gov/study/NCT05926297. The investigation was conducted at the Department of Periodontology and Implantology, University of Naples Federico II from July 2023 to December 2024.

### 2.2 Sample Size Calculation

The present study was designed to test a continuous response variable (i.e., change in mean BOP) from independent control and experimental patients with one control per experimental subject. In data proceeding from a previous study (Iorio‐Siciliano et al. 2020), at 1 month, the response within each subject was normally distributed with a standard deviation of 5.216. If the true difference in the experimental and control means is 5.095%, a sample of 34 patients (17 patients per group) is needed to reject the null hypothesis that the means of the sample size of the experimental and control groups are equal with probability (power) 0.8. The Type I error probability associated with this test of this null hypothesis is 0,05.

To avoid an underpowered sample size due to potential dropouts, a total of 20 patients with 20 implants were enrolled in each group. Sample size calculation was performed using a computer software (IBM‐SPSS, IBM Inc.).

### 2.3 Patient Enrollment

An initial screening was performed at the Dental School, University of Naples Federico II and subjects with implant‐supported dental prostheses diagnosed with PM were referred to the Department of Periodontology and Implantology, University of Naples Federico II for treatment. On the basis of the Implant Dentistry Core Outcome Set and Measurements (ID‐COSM) international consensus report (Tonetti et al. 2023), implants showing presence of bleeding (i.e., more than one spot at a location around the implant or presence of a line of bleeding or profuse bleeding at any location) and/or suppuration on gentle probing were diagnosed with PM. After initial screening, subjects who met the following eligibility criteria were progressively recruited. Once the sample size of 40 patients was obtained, the recruitment was stopped.

### 2.4 Eligibility Criteria

#### 2.4.1 Inclusion Criteria

The inclusion criteria were:

– Male and female ≥ 18 years.
– Patients with only a single implant supporting a cemented or screw‐retained single unit crown diagnosed with PM (Berglundh et al. 2018; Tonetti et al. 2023).
– Non‐smokers and smokers (≤ 10 cigarettes/day).
– Implant placed in mandible and in maxilla.
– Presence of at least 2 mm of keratinized attached mucosa at buccal and oral sites.

The diagnosis of peri‐implant mucositis required presence of bleeding and/or suppuration on gentle probing and absence of bone loss (Berglundh et al. 2018). Following the modification of the Implant Dentistry Core Outcome Set and Measurement (ID‐COSM) international consensus report, this definition was updated as presence of extensive BoP (≥ 2 spots/implant or ≥ 1 site/implant with a line or profuse bleeding) and/or suppuration on gentle probing (Tonetti et al. 2023). To exclude implants with peri‐implantitis, a radiograph was taken for all implants only at baseline.

#### 2.4.2 Exclusion Criteria

The exclusion criteria were as follows:

– Patients with systemic diseases.
– Patients with 2 or more implants affected by PM.
– Patients with untreated periodontitis.
– Pregnant and lactating females.
– Use of antibiotics or inflammatory drugs within 3 months prior to study recruitment.
– Implants with modified necks or abutments (i.e., micro‐rough).
– Implant‐supported reconstructions with poor fit (i.e., detectible marginal gap).
– Peri‐implantitis (Berglundh et al. 2018; Renvert et al. 2018).

### 2.5 Outcome Measures

The primary outcome was the change in mean BoP (Renvert et al. 2018).

The following secondary clinical outcomes were recorded:

– Full‐Mouth Plaque Score (FMPS), representing the percentage of sites with plaque (O'Leary et al. 1972).
– Full‐Mouth Bleeding Score (FMBS) representing the percentage of sites showing bleeding on probing (Claffey et al. 1990).
– Presence of plaque according to the plaque index (PlI) (Silness and Loe 1964) and to the modified index for oral implants (mPI) (Mombelli et al. 1987).
– Probing depth (PD), vertical distance measured from the mucosal margin to the bottom of the peri‐implant sulcus.

**FIGURE 1:**
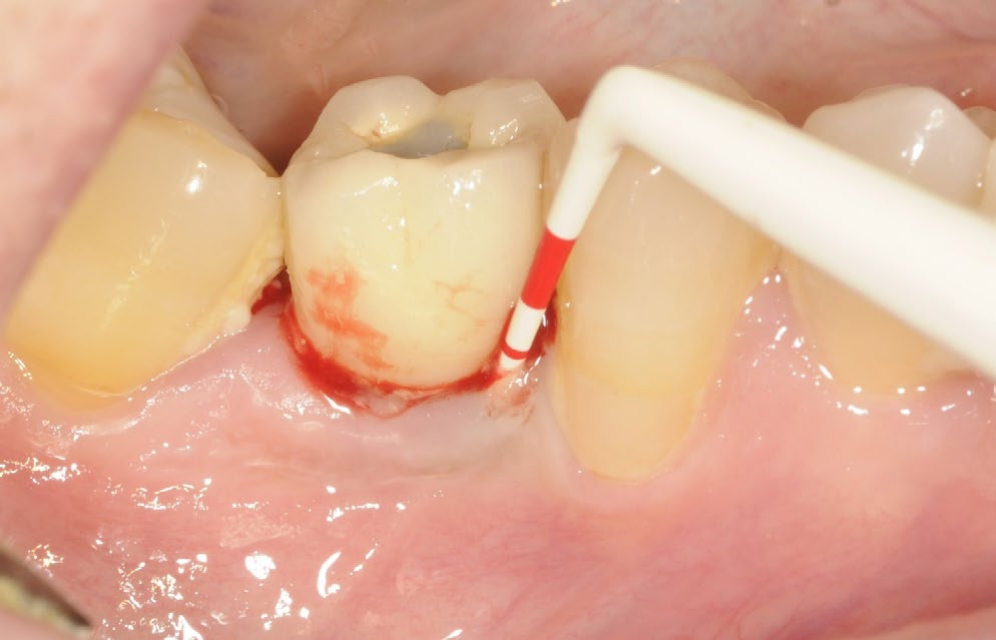
Single implant supporting screw‐retained single unit crown affected by PM.

Furthermore, disease resolution defined as implants without BoP or with the presence of a single bleeding spot (1 site/implant without a continuous line or profuse bleeding) was also recorded (Tonetti et al. 2023). The BoP was recorded in a dichotomous fashion (yes/no) (Figure 1). All clinical parameters were assessed at 6 sites/implant at baseline and after 1‐, 3‐, and 6‐month follow‐up. Clinical parameters were recorded by an experienced examiner (M.L.) using a manual periodontal probe (PerioWise color coded probe, Premier, Plymouth Meeting, PA, USA) applying a probing force of approximately 0.20–0.25 N. All data were collected at the Department of Periodontology and Implantology, University of Naples Federico II, Naples, Italy.

### 2.6 Randomization and Blinding

A simple randomization without restrictions using 1:1 allocation ratio was performed to randomly assign the patients to test or control procedure. The random allocation sequence was made by a clinician not involved in the study. The allocation concealment was carried out using numbered cards closed in an opaque envelope. The even numbers were associated to test group and odd number to the control group. Treatment allocation was performed immediately before treatment by opening the envelope containing the information. Implants allocated in test group received the adjunctive experimental therapy, whereas no adjunctive agents were delivered to control implants. A computerized random number generator (www.random.org) was used to perform the randomization.

The examiner of clinical parameters (M.L.) and the statistician (B.A.) were masked with respect to test and control procedures. On the contrary, clinicians performing the treatment and patients were not blinded.

### 2.7 Investigator Calibration

Examiner (M.L.) attended two calibration sessions on a total of 10 patients not involved in the study. During the first calibration session, repeated measurements for PD at six sites for each implant were performed once and BoP was assessed. The second calibration session was repeated after 7 days. The Cohen's kappa coefficient was used to verify the intra‐examiner agreement. A value of 0.825 and 0.615 was observed for the PD and BoP, respectively.

### 2.8 Intervention

**FIGURE 2:**
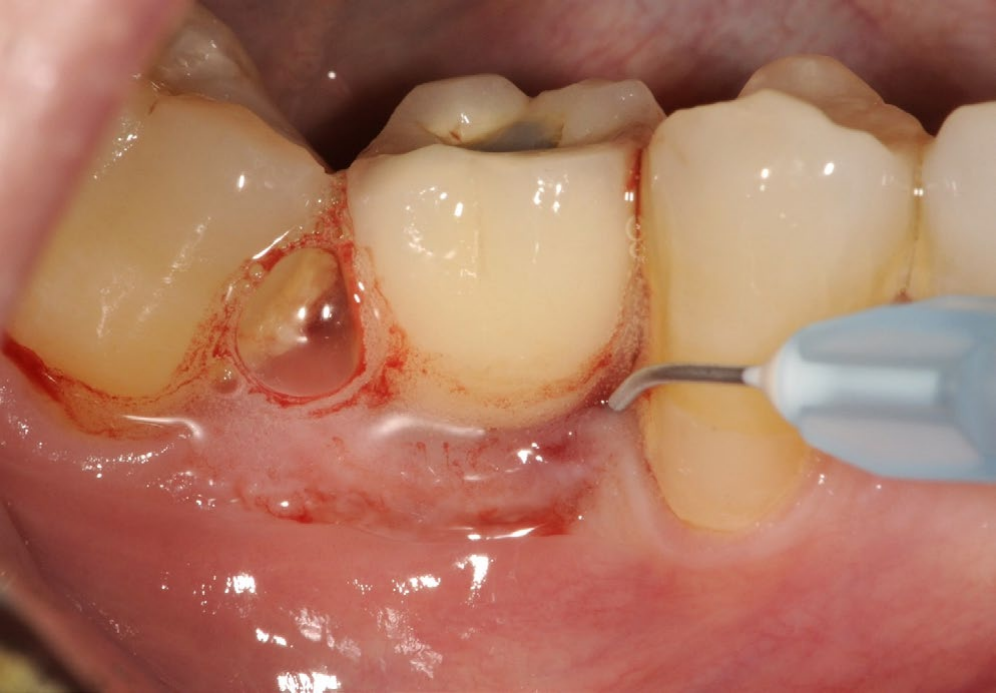
Local application of NaOCl 0.95%/amino acids.

**FIGURE 3:**
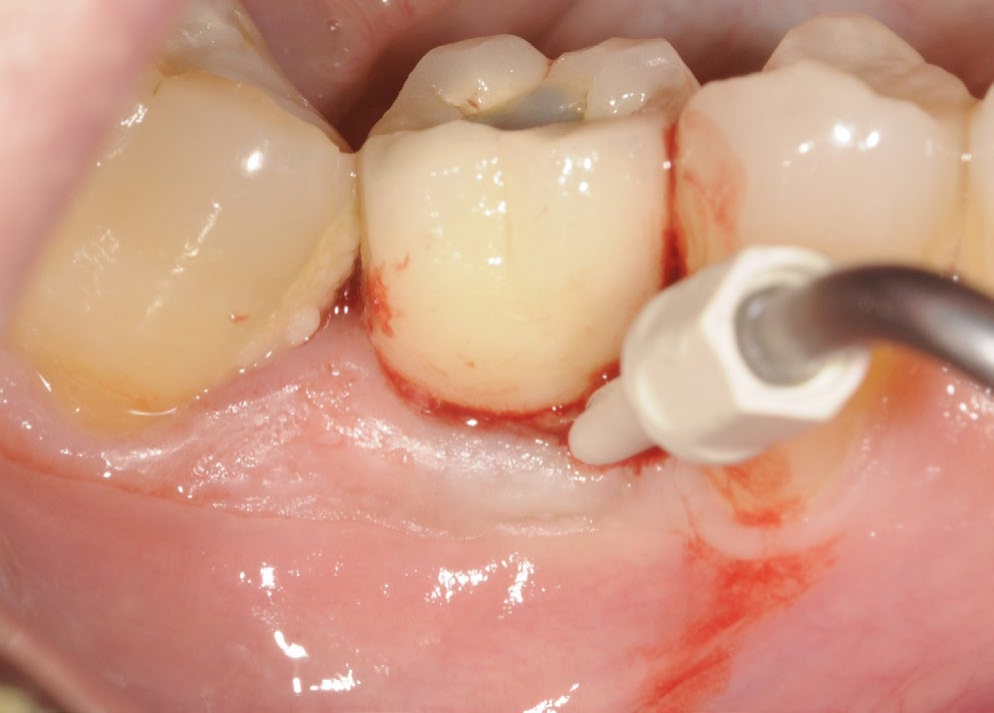
Submarginal instrumentation using sonic scaler with plastic tip.

**FIGURE 4:**
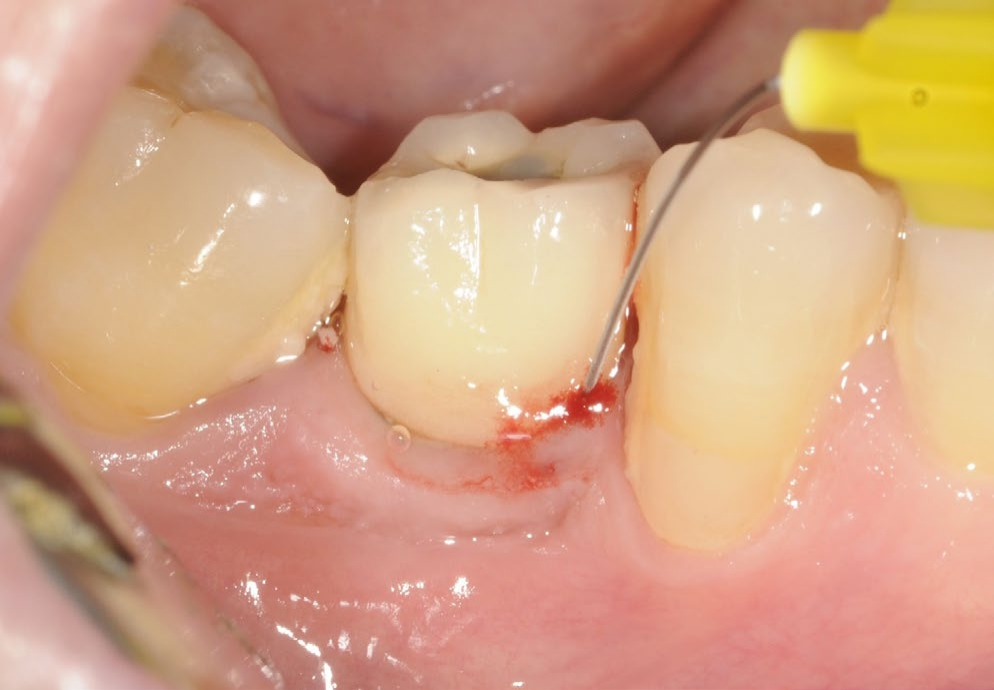
Local application of xHyA.

**FIGURE 5:**
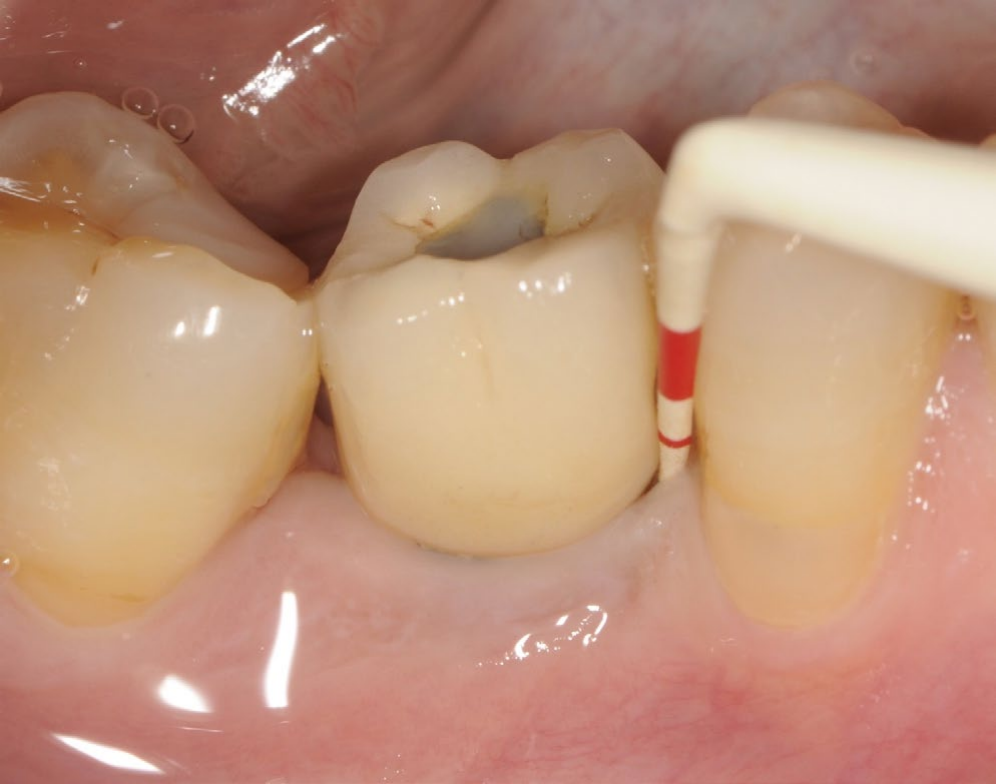
Clinical parameters assessed at 6 months.

Prior to treating the implant affected by PM, a full‐mouth SI using an ultrasonic device with metal tips was made in each patient. To facilitate biofilm removal, NaOCl 0.95%/amino acids gel (Perisolv, Regedent, Zürich, Switzerland) was applied in the peri‐implant sulcus to cover the full circumference of the implant (Figure 2). NaOCl 0.95%/amino acids gel was left in situ for 60 s. Afterwards, SI was performed using a sonic scaler with plastic tip (Kavo, SONICflex, Biberach, Germany) (Figure 3). After SI, the peri‐implant sulcus was rinsed with sterile saline solution. According to manufacturer's instructions, these procedures (e.g., NaOCl 0.95%/amino acids gel application and SI) were repeated 5 times around test implants in the same session. At the end of treatment, the peri‐implant sulcus was filled once using xHyA gel (Hyadent BG, Regedent, Zürich, Switzerland) (Figure 4). The control implants were treated with SI alone. After completion of both procedures, oral hygiene instructions were reinforced, and no antiseptic mouthwash was prescribed for either group. All clinical treatments were performed by the same expert operator (V.I.‐S.). All patients were recalled at 1‐, 3‐, and 6‐months following treatment for oral hygiene reinforcement and motivation (Figure 5). During the follow‐up no additional SI or application of NaOCl 0.95%/amino acids and xHyA was performed.

### 2.9 Statistical Analysis

Data analysis was performed using a statistical software package (IBM‐SPSS, IBM Inc.) and the statistician (B.A.) was masked with respect to the research protocol. The variables FMPS, FMBS, BoP, and mPlI were expressed in percentages, whereas PD was expressed in millimeters. For the parameters mPlI and BoP scores, the number of implants was also reported. An implant displaying at least 2 sites BoP‐positive or presence of a line of bleeding or profuse bleeding at any location was considered as BoP‐positive.

Data of patients lost during the follow‐up was not included and a per‐protocol analysis was carried out. Means and standard deviations, as well as median values and 95% CI, were calculated for each parameter except the number of implants with BoP, and mPlI. The percentage of BoP‐positive sites per implant was estimated at all time points.

Since each patient contributed only one implant to the study, the patient was considered as the statistical unit.

A non‐Gaussian specification was used within the framework of the generalized estimating equation (GEE) that was carried out in order to assess a correlation between group and differences in percentages of BoP‐positive sites per implant among longitudinal evaluations, and between group and changes in percentages of BoP‐positive sites per implant; the association was expressed as odds ratio (OR) with corresponding confidence intervals.

Differences in gender, smoking habit, implant location and type of prosthesis (i.e., cemented or screw‐retained) between test and control group were compared by means of a chi‐square test, whereas the Mann–Whitney *U* test was used for evaluating age. Inter‐group comparisons of FMPS, FMBS at 1‐, 3‐, and 6‐month follow‐ups were made using *U* Mann–Whitney test.

Intra‐group comparison for FMPS, FMBS, assessed at different time points was evaluated using Friedman and Wilcoxon's tests.

Inter‐group differences in terms of numbers of implants with plaque (mPli) and numbers of implants at 1‐, 3‐, and 6‐month follow‐ups were assessed with chi‐square test, whereas *U* Mann Whitney test was used for PD.

Treatment success (i.e., disease resolution) between test and control procedures at 1‐, 3‐, and 6‐month follow‐ups was compared using chi‐square test.

Furthermore, a comparison for treatment success using the *U* Mann–Whitney test was also performed stratifying implants on the basis of initial PD values (i.e., PD ≤ 3 mm, PD = 4 mm, PD = 5 mm).

Intra‐group comparison for PD assessed at different time points was evaluated using Friedman and Wilcoxon's tests while Cochran's *Q* and McNemar's test were used for mPlI, BoP‐positive implants, and treatment success (i.e., overall and stratified).

Number needed to treat (NNT) was also evaluated, calculating the reciprocal of the absolute risk difference between groups at 1‐, 3‐, and 6‐month follow‐ups (Altmann 1998).

A GEE was carried out in order to assess a correlation between predictors and treatment success among longitudinal evaluations. Since data were non‐normally distributed, a non‐Gaussian specification was used within the GEE framework; the association was expressed as odds ratios with corresponding confidence intervals.

All data were collected and analyzed at the Department of Periodontology, University of Naples Federico II, Naples, Italy. A *p*‐value < 0.05 was considered statistically significant.

## 3 Results

### 3.1 Patient Population

**FIGURE 6:**
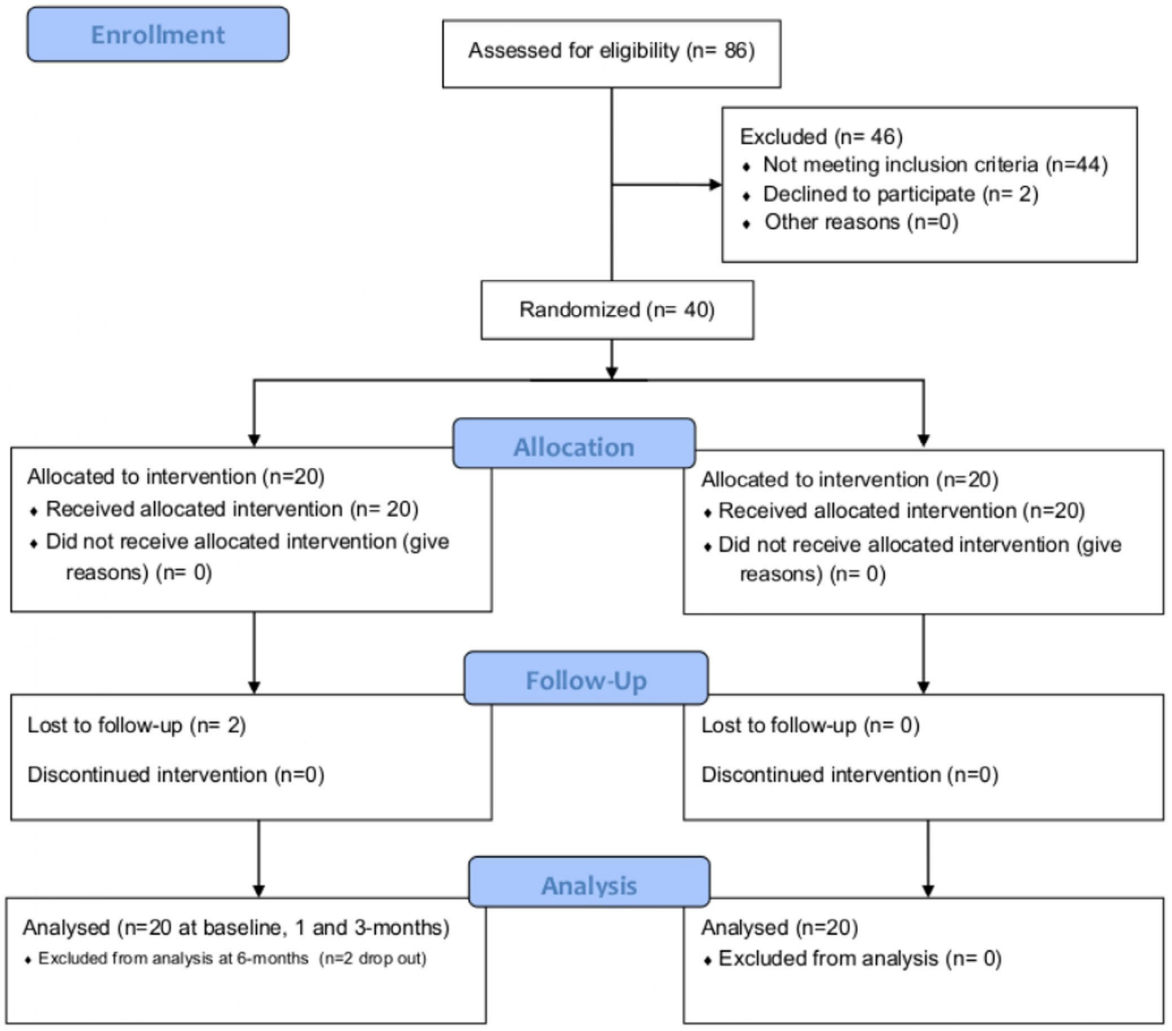
Flow diagram.

A total of 86 patients with one or more implants diagnosed with PM (Berglundh et al. 2018; Tonetti et al. 2023) were screened. After an initial screening, 44 subjects did not meet the inclusion criteria, whereas 2 patients declined to participate in the trial. A total of 40 patients with 40 implants affected by PM meeting the inclusion criteria were enrolled and randomly assigned to test or control procedure. At 4 months, 2 drop‐outs were recorded in the test group. Finally, 38 patients completed the study (Figure 6). During the investigation, no adverse events were recorded and no implants were lost.

### 3.2 Patient Characteristics

The characteristics of the participants are illustrated in Table 1. Ten females and 10 males (mean age 51.1 ± 11.7 years) were allocated to the test group, whereas 12 males and 8 females (mean age 50.3 ± 8.8 years) were assigned to the control group.

**TABLE 1:** Patient population and implant location at baseline.

	Test group (*n* = 18)	Control group (*n* = 20)	Significance
Gender (M/F)	9/9	12/8	0.745*
Age range (years) min–max	26–80	37–67	
Mean age (years)	52.0 ± 15.1	50.3 ± 8.8	0.829*
Smoking habit (Y/N)	5/13	5/15	0.999*
Implant location (A/P)	7/11	4/16	0.288*
Type of prosthetic reconstruction (C/S)	7/11	13/7	0.193*

Abbreviations: A, anterior area; C, cemented; F, female; M, male; N, no; P, posterior area; S, screwed; Y, yes.

* No statistically significant difference.

Five patients in each group were tobacco smokers (≤ 10 cigarettes/day). In the test group, 8 implants were located in the anterior area and 12 in the posterior area, whereas 4 and 16 implants were placed in the anterior and posterior areas of the control group. Twelve implants in the test group and 7 implants in the control group were rehabilitated by means of screw‐retained crowns, whereas 8 prosthetic reconstructions in the test group and 13 in the control group were cemented. No statistically significant differences were found between groups (*p* > 0.05) (Table 1).

### 3.3 FMPS and FMBS

FMPS and FMBS significantly improved in both groups from baseline to 6‐month follow‐up (*p* < 0.05). At 6 months, FMPS changed from 55.7% ± 6.5% to 19.5% ± 1.2% in the test group, whereas FMPS decreased from 49.6% ± 7.2% to 17.3% ± 2.9% in the control group. Likewise, a FMBS reduction from 48.9% ± 9.6% to 16.4% ± 3.7% and from 50.2% ± 4.2% to 18.2% ± 1.7% was noted in the test and control groups, respectively. Inter‐group comparison did not show statistically significant differences (*p* > 0.05) (Table 2).

**TABLE 2:** FMPS and FMBS at baseline, 1‐, 3‐, and 6‐month follow‐up.

	Baseline	1 month	3 months	6 months	Significance
FMPS (%)					
Test group (*n* = 18)					
Mean ± SD	55.7 ± 6.5^†,‡,§^	16.7 ± 2.9^Ҩ,§^	17.6 ± 2.1^Ҩ,§^	19.5 ± 1.2^Ҩ,†,‡^	0.0001**
Median (min–max)	56.5 (40.0–72.0)	16.5 (11.0–21.0)	17.0 (13.0–20.0)	19.0 (16.0–20.0)	
Standard error	1.9	0.8	0.6	0.2	
Control group (*n* = 20)					
Mean ± SD	49.6 ± 7.2^†,‡,§^	14.7 ± 2.9^Ҩ,§^	15.3 ± 2.4^Ҩ,§^	17.3 ± 2.9^Ҩ,†,‡^	0.0001**
Median (min–max)	57.5 (45.0–63.0)	17.0 (11.0–20.0)	18.5 (14.0–20.0)	20.0 (19.0–24.0)	
Significance		0.249*	0.518*	0.405*	
FMBS (%)					
Test group (*n* = 18)					
Mean ± SD	48.9 ± 9.6^†,‡,§^	14.2 ± 3.5^Ҩ^	14.3 ± 2.6^Ҩ,§^	16.4 ± 3.7^Ҩ,‡^	0.0001**
Median (min–max)	50.0 (25.0–65.0)	14.0 (7.0–19.0)	14.5 (10.0–19.0)	18.0 (8.0–20.0)	
Control group (*n* = 20)					
Mean ± SD	50.2 ± 4.2^†,‡,§^	15.2 ± 2.3^Ҩ,§^	16.15 ± 1.8^§^	18.2 ± 1.7^Ҩ,†,‡^	0.0001**
Median (min–max)	50.0 (40.0–59.0)	15.5 (10.0–18.0)	16.0 (14.0–19.0)	18.5 (15.0–22.0)	
Significance		0.425*	0.117*	0.357*	

Abbreviations: CI, confidence interval; FMBS, Full‐Mouth Bleeding Score; FMPS, Full‐Mouth Plaque Score; SD, standard deviation.

* No statistically significant difference.

** Statistically significant difference: ^Ҩ^vs. baseline; ^†^vs. 1 month; ^‡^vs. 3 months; ^§^vs. 6 months.

### 3.4 Change in Mean BoP (Primary Outcome)

The percentages and variations (i.e., mean BoP change) of number of BoP‐positive sites per implant are summarized in Table 3.

**TABLE 3:** Percentages and variations (BoP‐change) of numbers of BOP‐positive sites per implant at baseline, 1‐, 3‐, and 6‐months follow‐up.

BoP (%)							
	Baseline	1 month	3 months	6 months	Δ Bl 1‐month	Δ Bl 3‐months	Δ Bl 6‐months
Test group (*n* = 18)							
Mean ± SD	92.6 ± 11.7	12.0 ± 19.6	14.8 ± 24.8	23.1 ± 16.7	80.6 ± 24.3	77.8 ± 24.9	69.4 ± 28.2
Median (min–max)	100.0 (66.7–100.0)	0.0 (0.0–20.8)	0.0 (0.0–50.0)	16.7 (0.0–66.7)	83.3 (50.0–100.0)	83.3 (33.3–100.0)	83.3 (16.7–100.0)
Control group (*n* = 20)							
Mean ± SD	88.3 ± 15.4	25.8 ± 18.3	28.3 ± 33.3	37.5 ± 28.0	62.5 ± 20.9	60.0 ± 21.9	50.8 ± 25.1
Median (min–max)	100.0 (50.0–100.0)	33.3 (0.0–66.7)	33.3 (0.0–66.7)	41.7 (0.0–83.3)	66.7 (16.7–100.0)	58.3 (33.3–100.0)	50.0 (16.7–100.0)

	Significance	Exp (*B*)	95% Wald's CI for Exp (*B*)	
			Lower	Upper
Generalized estimating equation (GEE) for percentages of BOP‐positive sites per implant				
Intercept	< 0.001**	3.49e^19^	1.70e^16^	7.20e^22^
Group (test vs. control)	0.089*	< 0.001	1.79e^−9^	4.20
Generalized estimating equation (GEE) for variations of percentages of BOP‐positive sites per implant				
Intercept	< 0.001**	6.598e^18^	8.80e^15^	4.94e^21^
Group (test vs. control)	0.006**	8.15e^5^	49.03	1.35e^10^

Abbreviations: BoP, bleeding on probing; CI, confidence interval; SD, standard deviation.

* No statistically significant difference.

** Statistically significant difference.

At baseline, the percentages of number of BoP‐positive sites per implant were 92.6% ± 11.7% in the test group and 88.3% ± 15.4% in the control group. After 1 month, these percentages were 12.0% ± 19.6% and 25.8% ± 18.3% in the test and control groups, respectively, whereas after 3 months the percentage of number of BoP‐positive sites per implant was 14.8% ± 24.8% in the test group and 28.3% ± 33.3% in the control group.

At 6 months, percentages of 23.1% ± 16.7% and 37.5% ± 28.0% were recorded in the test and control groups, respectively.

The mean BoP change (primary outcome) at 1, 3, and 6 months was 72.2% ± 24.3%, 70.4% ± 24.3%, and 63.0% ± 24.6% for the implants of the test group. On the contrary, a mean BoP change of 70.0% ± 19.9%, 66.7% ± 24.8%, and 56.7% ± 30.8% was recorded in the control group at 1, 3, and 6 months, respectively (Table 3).

No significant correlation between percentages of BoP positive sites per implant and allocation into test group was recorded. With regard to mean BoP change, instead, a significant correlation with group allocation was recorded with 8.15e^5^ OR.

### 3.5 Number of Implants With Plaque, Number of Implants With BoP‐Positive and Probing Depths

Table 4 reports the number and percentage of implants with plaque, implants BoP‐positive, and the means of PD changes. After 6 months following therapy, the number of implants with plaque reduced significantly in both groups (*p* < 0.05). Prior to starting with the treatments, the number of implants with plaque was 18 (100%) and 20 (100%) in the test and control groups, respectively. At 1 month, 5 (27.8%) implants of the test group and 11 (55.5%) of the control group showed visual plaque accumulation. At 3 months, the number of implants with plaque was 5 (27.8%) and 12 (60%) for the test and control groups, respectively, whereas 6 months following therapy of PM the number of implants with plaque was 4 (22.2%) in the test group and 12 (60%) in the control group.

**TABLE 4:** Changes in numbers of implants with plaque, number of implants with BoP‐positive and probing depths at baseline, 1‐, 3‐, and 6‐month follow‐up.

	Baseline	1 month	3 months	6 months	Significance	Δ Bl 1‐month	Δ Bl 3‐months	Δ Bl 6‐months	*p*
*N*/% of implants with Plaque (mPlI)									
Test group (*n* = 18)	18 (*n*)/100 (%)^†,‡,§^	5 (*n*)/27.8 (%)^Ҩ^	5 (*n*)/27.8 (%)^Ҩ^	4 (*n*)/22.2 (%)^Ҩ^	0.0001**	13 (*n*)/72.2 (%)	13 (*n*)/72.2 (%)	14 (*n*)/77.8 (%)	0.846*
Control group (*n* = 20)	20 (*n*)/100 (%)^†,‡,§^	11 (*n*)/55 (%)^Ҩ^	12 (*n*)/60 (%)^Ҩ^	12 (*n*)/60 (%)^Ҩ^	0.001**	9 (*n*)/45 (%)	8 (*n*)/40 (%)	8 (*n*)/40 (%)	0.846*
Significance		0.090*	0.046**	0.019**		0.484*	0.279*	0.134*	
*N*/% of implants with BoP‐positive									
Test group (*n* = 18)	18 (*n*)/100 (%)^†,‡,§^	4 (*n*)/22.2 (%)^Ҩ^	5 (*n*)/27.8 (%)^Ҩ^	5 (*n*)/27.8 (%)^Ҩ^	0.0001**	14 (*n*)/77.8 (%)	13 (*n*)/72.2 (%)	13 (*n*)/72.2 (%)	0.819*
Control group (*n* = 20)	20 (*n*)/100 (%)^†,‡,§^	11 (*n*)/55 (%)^Ҩ^	12 (*n*)/60 (%)^Ҩ^	12 (*n*)/60 (%)^Ҩ^	0.0001**	9 (*n*)/45 (%)	8 (*n*)/40 (%)	8 (*n*)/40 (%)	0.264*
Significance		0.039**	0.046**	0.046**		0.458*	0.279*	0.279*	
PD (mm)									
Test group (*n* = 18)									
Mean ± SD	4.2 ± 0.8^†^	3.4 ± 0.5^Ҩ,‡,§^	3.7 ± 0.5^†^	3.9 ± 0.3^†^	0.004**	0.8 ± 1.0^‡,§^	0.5 ± 1.1^†^	0.3 ± 0.9^†^	0.003**
Median (min–max)	4.0 (3.0–5.0)	3.0 (3.0–4.0)	4.0 (3.0–4.0)	4.0 (3.0–4.0)		1.0 (−1.0–2.0)	0.5 (−1.0–2.0)	0.5 (−1.0–2.0)	
Control group (*n* = 20)									
Mean ± SD	4.1 ± 0.9^†^	3.6 ± 0.5^Ҩ,§^	3.8 ± 0.4	4.0 ± 0.2^†^	0.025**	0.6 ± 1.0^§^	0.3 ± 0.9	0.2 ± 0.8^†^	0.012**
Median (min–max)	4.0 (3.0–5.0)	4.0 (3.0–4.0)	4.0 (3.0–4.0)	4.0 (3.0–4.0)		1.0 (−1.0 to 2.0)	0.0 (−1.0 to 2.0)	0.0 (−1.0 to 1.0)	
Significance		0.321*	0.573*	0.485*		0.426*	0.593*	0.553*	

Abbreviations: %, percentage; BOP‐positive, implants showing at least 2 sites with BOP‐positive; mPlI, modified plaque index; *n*, number; PD, probing depth.

* No statistically significant difference.

** Statistically significant difference: ^Ҩ^vs. baseline; ^†^vs. 1 month; ^‡^vs. 3 months; ^§^vs. 6 months.

No statistically significant changes were observed between test and control implants at baseline and after 3 months follow‐up (*p* > 0.05). On the contrary, statistically significant differences in favor of the test group were found at 1 and 6 months, respectively (*p* < 0.05).

At baseline, all implants were BoP‐positive (100%). The intra‐group analysis showed a statistically significant reduction in number and proportion of implants with BoP‐positive (*p* < 0.05) between baseline and follow‐ups. At 1 month, 4 (22.2%) implants of the test group and 11 (55.5%) implants of the control group were BoP‐positive, whereas 5 (27.8%) in the test group and 12 (60%) implants in the control group were BoP‐positive after 3‐month follow‐up. After 6 months, 5 (27.8%) and 12 (60%) were BoP‐positive. Statistically significant differences were noted in favor of the experimental procedure when test and control groups were compared at 1, 3, and 6 months (*p* < 0.05).

The probing depth (PD) significantly decreased between baseline and 6 months follow‐up (*p* < 0.05). At baseline, implants of the test group showed a PD of 4.2 ± 0.8 mm, whereas a PD of 4.1 ± 0.9 mm was recorded in the control group. At 1 month, the PD was 3.4 ± 0.5 and 3.6 ± 0.5 mm for the test and control implants, respectively. After 3 months, PD was 3.7 ± 0.5 mm for implants of the test group and 3.8 ± 0.4 mm for implants of the control group. At 6 months, a PD of 3.9 ± 0.3 and 4.0 ± 0.2 mm was assessed for implants of the test and control groups, respectively.

No statistically significant changes were noted between test and control group at 1‐, 3‐, and 6‐months (*p* > 0.05) (Table 4).

### 3.6 Disease Resolution (i.e., Treatment Success)

Table 5 shows the number and percentage of implants with a disease resolution (i.e., treatment success) following test and control procedures. One month after therapy, 14 (78.8%) of test implants and 9 (45%) of control implants achieved disease resolution (*p* < 0.05). After 3 months 13 (72.2%) implants of the test group and 8 (40%) implants of control group yielded resolution of PM (*p* < 0.05). At 6 months, disease resolution was observed in 13 (72.2%) implants of the test group, whereas in the control group 8 (40%) implants achieved complete healing (*p* < 0.05).

**TABLE 5:** Number and percentage of BoP‐negative implants (treatment success) at 1‐, 3‐, and 6‐months follow‐up.

BoP‐negative implants (*n*/%)					
	Test group (*n* = 18)	Control group (*n* = 20)	Significance	NNT	95% CI
1 month	14 (*n*)/78.8 (%)	9 (*n*)/45 (%)	0.039**	3.05	3.72–61.83
3 months	13 (*n*)/72.2 (%)	8 (*n*)/40 (%)	0.046**	3.1	2.40–62.04
6 months	13 (*n*)/72.2 (%)	8 (*n*)/40 (%)	0.046**	3.1	2.40–62.04
Significance	0.819*	0.779*			

Abbreviations: %, percentage; BoP, ≤ 1 site with BoP‐positive; *n*, number.

* No statistically significant difference.

** Statistically significant difference.

Intra‐group comparison between 1‐, 3‐, and 6‐months follow‐up did not show a statistically significant difference in terms of disease resolution (*p* > 0.05). On the contrary, statistically significant differences in favor of the experimental procedure were recorded between test and control group (*p* < 0.05) (Table 5). The number and percentages of implants with BoP‐negative (i.e., treatment success) at 1‐, 3‐, and 6‐months based on initial probing depth (PD) are illustrated in Table 6. The intra‐group analysis between 1‐, 3‐, and 6‐months did not show statistically significant differences in terms of disease resolution with respect to initial PDs (*p* > 0.05). Similarly, the comparison between test and control procedures of implants with initial PD ≤ 3 mm and PD = 4 mm did not yield significantly statistically differences at each follow‐up (*p* > 0.05). The resolution of PM was observed in 3 (75%) test and 5 (83.3%) control implants with initial PD ≤ 3 mm after 1‐, 3‐, and 6‐month follow‐up. Four test Implants (66.7%) and 3 (50%) control implants with an initial PD = 4 mm achieved disease resolution after 1‐month follow‐up, whereas at 3‐ and 6‐months complete healing resulted in 4 (66.7%) and 2 (33.3%) implants treated with test or control procedure, respectively. Otherwise, statistically significant differences (*p* < 0.05) were found comparing PM resolution of implants with initial PD = 5 mm treated with experimental procedure with respect to control procedure.

**TABLE 6:** Numbers and percentages of BoP‐negative implants (treatment success) at 1‐, 3‐, and 6‐month follow‐ups based on initial probing depth.

	BoP‐negative implants (*n*/%)				
		1 month	3 months	6 months	Significance
PD ≤ 3 mm	Test group (*n* = 4)	3 (*n*)/75 (%)	3 (*n*)/75 (%)	3 (*n*)/75 (%)	0.999*
	Control group (*n* = 6)	5 (*n*)/83.3 (%)	5 (*n*)/83.3 (%)	5 (*n*)/83.3 (%)	0.607*
	Significance	0.747*	0.747*	0.747*	
PD = 4 mm	Test group (*n* = 6)	4 (*n*)/66.7 (%)	4 (*n*)/66.7 (%)	4 (*n*)/66.7 (%)	0.999*
	Control group (*n* = 6)	3 (*n*)/50 (%)	2 (*n*)/33.3 (%)	2 (*n*)/33.3 (%)	0.368*
	Significance	0.334*	0.248*	0.248*	
PD = 5 mm	Test group (*n* = 8)	7 (*n*)/87.5 (%)	6 (*n*)/75 (%)	6 (*n*)/75 (%)	0.717*
	Control group (*n* = 8)	1 (*n*)/12.5 (%)	1 (*n*)/12.5 (%)	1 (*n*)/12.5 (%)	0.999*
	Significance	0.003**	0.012**	0.012**	

Abbreviations: %, percentage; BoP, ≤ 1 site with BoP‐positive; *n*, number; PD, probing depth.

* No statistically significant difference.

** Statistically significant difference.

Seven (87.5%) implants in the test group and 1 (12.5%) implant of the control group were BoP‐negative after 1‐month follow‐up. At 3 and 6 months, resolution of PM was recorded in 6 (75%) implants of the test group and in 1 implant of the control group, respectively (Table 6).

The data of GEE for treatment success are presented in Table 7. A significant correlation was found for the group (i.e., test vs. control) (*p* < 0.05) with an exp (*B*) of 3.77 (Table 7).

**TABLE 7:** Generalized estimating equation (GEE) for treatment success.

	Significance	Exp (*B*)	95% Wald's CI for Exp (*B*)	
			Lower	Upper
Intercept	0.766*	0.617	0.026	14.706
Group (test vs. control)	0.036**	3.777	1.090	13.087
Gender (male vs. female)	0.699*	0.778	0.217	2.785
Age	0.696*	0.990	0.940	1.042
Smoking habit (yes vs. no)	0.359*	2.035	0.445	9.297
Type of prosthetic reconstruction (cemented vs. screwed)	0.773*	0.8016	0.204	3.256

* No statistically significant difference.

** Statistically significant difference.

## 4 Discussion

The present study compared the healing of PM treated by means of SI with or without local application of NaOCl 0.95%/amino acids and xHyA compared with SI alone. After 6 months follow‐up, the implants of both groups showed an improvement in clinical parameters and statistically significant differences in terms of change in mean BoP (i.e., primary outcome) were recorded between test and control treatment. Based on these results, the null hypothesis of no statistically significant differences between test and control procedures with respect to the change in mean BoP was rejected. The outcomes of present study are in partial agreement with those reported in previous studies on the treatment of PM using adjunctive topical antiseptic agents. The evidence suggested that mechanical cleaning alone may be effective to treat PM (i.e., BoP reduction), and adjunctive delivery of antiseptics (i.e., irrigations or gel) did not enhance the results as compared to mechanical debridement alone (Ramanauskaite et al. 2021). Likewise, in our study, the use of NaOCl 0.95%/amino acids and xHyA as an adjunct to PMPR resulted in no statistically significant differences with respect to control procedures overtime in terms of percentages of BoP‐positive sites per implant. However, in the present study a statistically significant difference in BoP reduction was observed overtime between test and control group. On the contrary, Menezes and co‐workers did not record statistically significant differences using an adjunctive antiseptic (i.e., chlorhexidine gel) with respect to mechanical debridement alone, and the BoP reduction was only 30% (Menezes et al. 2016). Although the clinical relevance of the superiority observed in the present study should be interpreted with caution and independently according to the clinician's point of view, potential explanations may be related to the capacity of NaOCl 0.95%/amino acids to facilitate the plaque removal penetrating the biofilm (Brown and Gilbert 1993) and to the anti‐inflammatory proprieties of the xHyA (Sasaki and Watanabe 1995). At 6‐months, both clinical procedures resulted in PD reduction less than 1 mm. Certainly, the PD reduction depended on the limited mucosal recession as result of PM resolution and soft tissue healing. In any case, PD reduction cannot be considered the endpoint of PM therapy (Renvert et al. 2018), since the probing depth at implant sites depends on many factors (i.e., high of the soft tissues at implant location).

In both groups, FMPS and FMBS increased gradually during the follow‐up. High FMPS and FMBS could negatively influence the results of the primary outcome. In a previous study (Isola et al. 2025), a multi‐level regression model evidenced that a high FMPS, FMBS, and concentration of *P. gingivalis* and *T. forsythia* significantly influenced the BoP reduction after non‐surgical treatment of PM with or without adjunctive delivery.

However, in our investigation, FMPS and FMBS were below 20% at 1‐ and 6‐month follow‐up, indicating that periodontal conditions were continuously well maintained.

In the present investigations, test and control implants achieved a complete disease resolution (i.e., treatment success) of 72.2% and 40% after 6‐months follow‐up, and statistically significant differences were observed between groups. These results are in contrast with those of previous studies on treatment of PM using local antiseptic application in combination with PMPR. Heitz‐Mayfield et al. (2011) obtained a 38% of complete disease resolution following PM treatment with PMPR and local application of chlorhexidine gel, whereas Iorio‐Siciliano et al. (2020) achieved a 45% of complete PM healing using the combination of PMPR and NaOCl 0.95%/amino acids. On the contrary, similar results (i.e., 70% complete disease resolution) were found using a formulation of spermidine and sodium hyaluronate associated to PMPR in the management of PM (Iorio‐Siciliano et al. 2024). Probably, the improvement of clinical parameters of the present trial depends on the effect of xHyA rather than on the application of the antiseptic agents. Indeed, preclinical outcomes demonstrated that xHyA triggers the expression of prominent indicators of wound healing (i.e., TGF β3) and upregulates the expression of genes encoding the growth factors (PDGFB, FGF‐2) essential for proper wound healing (Asparuhova et al. 2019). Nevertheless, the limited effect of NaOCl 0.95%/amino acids noted by Iorio‐Siciliano et al. (2020) depend on the application time (i.e., 30 s). In the present study, the NaOCl 0.95%/amino acids was delivered to 60 s in the peri‐implant sulcus prior to start with PMPR. The prolonged application time increases the capacity of NaOCl 0.95%/amino acids to penetrate the biofilm facilitating the removal (Ramanauskaite et al. 2023).

The high percentages of treatment success achieved in the current study with respect to those of previous studies must be interpreted with caution, since a different definition of complete disease resolution was used. Previously, treatment success (i.e., PM resolution) was defined as complete absence of BoP assessed at 4 or 6 sites around implant, whereas in the present trial an implant presenting with complete absence of BoP or with the presence of a single bleeding spot (i.e., 1 site/implant without a continuous line or profuse bleeding) was considered as healed (Tonetti et al. 2023). A relevant result of the present study is strong correlation between treatment success and allocation into test group. This result reflects the percentage (27.8%) of implants displaying bleeding in test group with respect to those in control group (60.0%).

The subgroup analysis reported that complete disease resolution of implants with an initial PD ≤ 4 mm did not differ significantly when test and control procedures were compared, whereas statistically significant differences were found for implants with an initial PD of 5 mm.

Although these percentages referred to different implants at each time point, the number of resolved cases did not differ across time point (1, 3, and 6 months).

These data indicated that PMPR alone (i.e., control procedure) seemed ineffective in BoP reduction for implants with a deep peri‐implant sulcus (i.e., PD = 5 mm). Similar results were reported in a previous investigation conducted by Chan et al. (2019) on the influence of depth of mucosal tunnel in the resolution of experimental PM. These authors reported significant changes in clinical parameters and in peri‐implant sulcus fluid IL‐1β concentrations comparing implants with deep mucosal tunnel (i.e., ≥ 3 mm) and implants with shallow mucosal tunnel (i.e., ≤ 1 mm). Furthermore, the probability for a peri‐implant site to be BoP‐positive increases with increasing PD (i.e., OR increased by 1.6 for each 1 mm increment in PD) (Farina et al. 2017). For these reasons, the local application of NaOCl 0.95%/amino acids and xHyA as an adjunct to SI would seem to be beneficial in the treatment of implants affected by PM with an initial PD of 5 mm. However, these outcomes need to be confirmed by further studies due to the limited sample size of each subgroup.

In the present study many reconstructions were cemented (i.e., 7 in the test group and 13 in the control group). Although clinical parameters (i.e., BoP changes) improved after removal of screw‐retained crowns and submucosal instrumentation in the study by Chan et al. (2019), submucosal professional cleaning was performed without removal of the screw‐retained crowns in the present study to avoid a treatment bias. For the same reason, the initial probing depth and not the depth of the mucosal tunnel (i.e., distance measured from the implant shoulder to the mucosal margin) was assessed. Certainly, initial PD and mucosal tunnel cannot be considered similar parameters, and many factors influence the PD recording (i.e., soft tissue thickness, presence of prosthetic reconstruction). This aspect could represent a further limitation of the present study. Finally, lack of patient‐reported outcomes (i.e., PROMs) represented another limitation of the present study. In fact, the anti‐inflammatory proprieties of the xHyA may reduce the post‐operative disconfort.

In conclusion, within the limitations of this study, the findings indicated that professional local delivery of NaOCl 0.95%/amino acids and xHyA lead to additional clinical benefits when used as an adjunct to SI in the management of PM compared with SI alone. Disease resolution, however, was not achieved in both treatment groups.

## Author Contributions

V.I.‐S. treated the patients, A.B. performed the statistical analysis, L.M. patient enrollment and data acquisition, P.W. co‐drafted the manuscript, A.S. co‐drafted the protocol and manuscript, G.E.S. co‐drafted the manuscript and data interpretation, and L.R. project administration.

## Funding

The study was supported by the University of Naples Federico II funds.

## Conflicts of Interest

The authors declare no conflicts of interest.

## Supporting information


**Appendix S1:** clr70109‐sup‐0001‐AppendixS1.pdf.

## Data Availability

The data that support the findings of this study are available on request from the corresponding author. The data are not publicly available due to privacy or ethical restrictions.
