# Supplementary material for: Non‐Surgical Submarginal Instrumentation of Peri‐Implant Mucositis With Delivery of Sodium Hypochlorite/Amino Acids and Cross‐Linked Hyaluronic Acid: A Randomized Clinical Trial
Source: Clin Oral Implants Res. 2026 Feb 27;37(5):643–56. doi: 10.1111/clr.70109 (PMC13155298; doi:10.1111/clr.70109)
Supplement: Supplementary file 1 — Appendix S1: clr70109‐sup‐0001‐AppendixS1.pdf. [file CLR-37-643-s001.pdf]

Explore

Notes

Output Created		22-JAN-2026 16:22:56
Comments		
Input	Data	C:\Users\Andrea\Documents\2022\Enzois\statisticacoir_mucositi\Dati_nodropout.sav
	Active Dataset	Dataset1
	Filter	<none>
	Weight	<none>
	Split File	<none>
	N of Rows in Working Data File	152
Missing Value Handling	Definition of Missing	User-defined missing values for dependent variables are treated as missing.
	Cases Used	Statistics are based on cases with no missing values for any dependent variable or factor used.
Syntax		EXAMINE VARIABLES=BOP_PERC_SITES BY GROUP1T2C /ID=VISIT /PLOT BOXPLOT STEMLEAF HISTOGRAM NPPLOT /COMPARE GROUPS /STATISTICS DESCRIPTIVES /CINTERVAL 95 /MISSING LISTWISE /NOTOTAL.
Resources	Processor Time	00:00:03,17
	Elapsed Time	00:00:01,13

```
[Dataset1] C:\Users\Andrea\Documents\2022\Enzois\statisticacoir_mucositi\Dati_nodropout.sav
```

GROUP1T2C

Case Processing Summary

	GROUP1T2C	Cases				
		Valid		Missing		Total
		N	Percent	N	Percent	N
BOP_PERC_SITES	1	72	100,0%	0	0,0%	72
	2	80	100,0%	0	0,0%	80

Case Processing Summary

	GROUP1T2C	Cases
		Total
		Percent
BOP_PERC_SITES	1	100,0%
	2	100,0%

Descriptives

	GROUP1T2C			Statistic	Std. Error
BOP_PERC_SITES	1	Mean		35,6481	4,67731
		95% Confidence Interval for Mean	Lower Bound	26,3219	
			Upper Bound	44,9744	
		5% Trimmed Mean		34,0535	
		Median		16,6667	
		Variance		1575,161	
		Std. Deviation		39,68830	
		Minimum		,00	
		Maximum		100,00	
		Range		100,00	
		Interquartile Range		66,67	
		Skewness		,564	,283
		Kurtosis		-1,338	,559
	2	Mean		45,0000	3,70786
		95% Confidence Interval for Mean	Lower Bound	37,6197	
			Upper Bound	52,3803	
		5% Trimmed Mean		44,4444	
		Median		41,6667	
		Variance		1099,859	
		Std. Deviation		33,16413	
		Minimum		,00	
		Maximum		100,00	
		Range		100,00	
		Interquartile Range		50,00	
		Skewness		,270	,269
		Kurtosis		-1,059	,532

Tests of Normality

	GROUP1T2C	Kolmogorov-Smirnov[a] Statistic	df	Sig.	Shapiro-Wilk Statistic	df
BOP_PERC_SITES	1	,260	72	<,001	,785	72
	2	,138	80	<,001	,914	80

Tests of Normality

	GROUP1T2C	Shapiro-... Sig.
BOP_PERC_SITES	1	<,001
	2	<,001

a. Lilliefors Significance Correction

BOP_PERC_SITES

Histograms

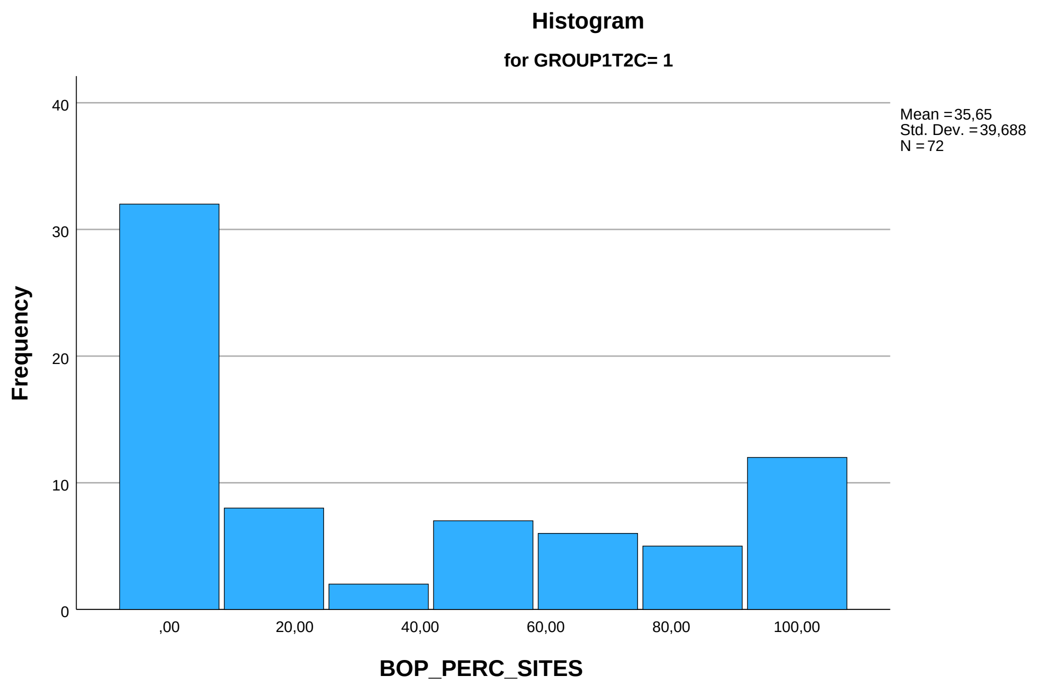

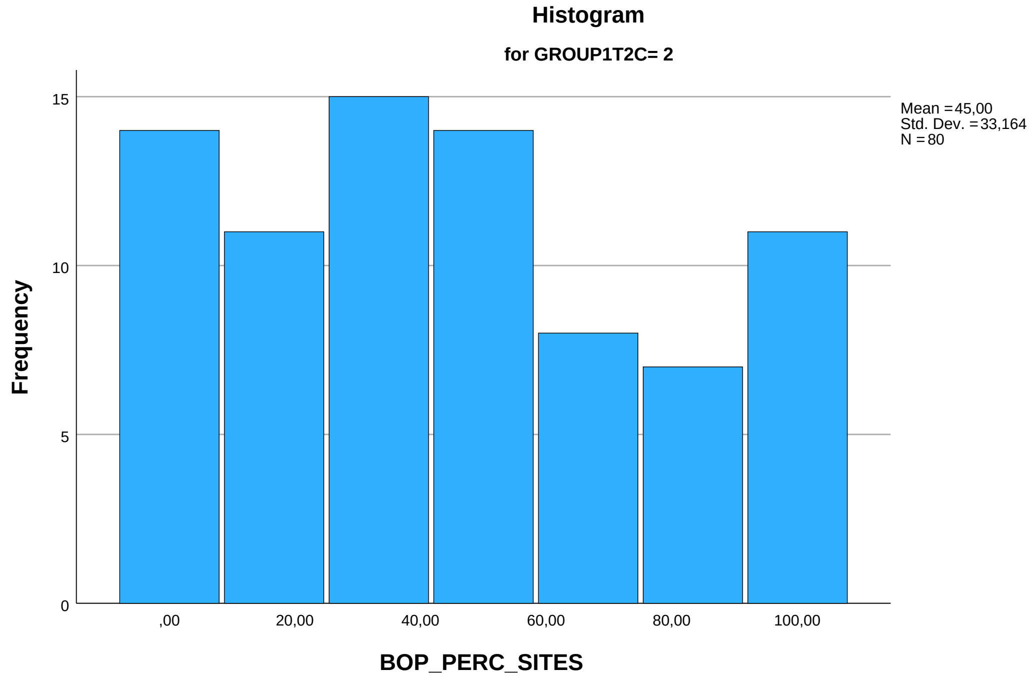

Stem-and-Leaf Plots

```
BOP_PERC_SITES Stem-and-Leaf Plot for
GROUP1T2C= 1

 Frequency    Stem &  Leaf

    40,00        0 .  0000000000000000000000000000000011111111
     2,00        0 .  33
     7,00        0 .  5555555
     6,00        0 .  666666
     5,00        0 .  88888
    12,00        1 .  000000000000

 Stem width:    100,00
 Each leaf:        1 case(s)
```

```
BOP_PERC_SITES Stem-and-Leaf Plot for
GROUP1T2C= 2

 Frequency    Stem &  Leaf

    25,00        0 .  0000000000000001111111111
    15,00        0 .  333333333333333
    14,00        0 .  55555555555555
     8,00        0 .  66666666
     7,00        0 .  8888888
    11,00        1 .  00000000000
```

```
Stem width:    100,00
Each leaf:        1 case(s)
```

Normal Q-Q Plots

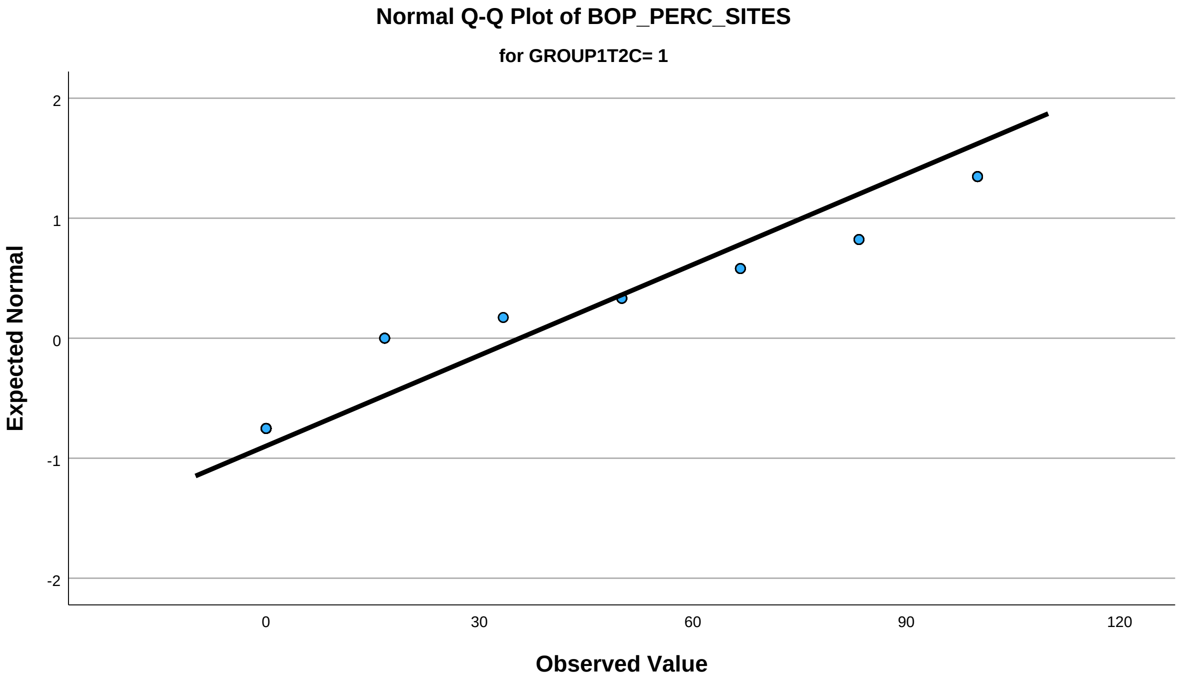

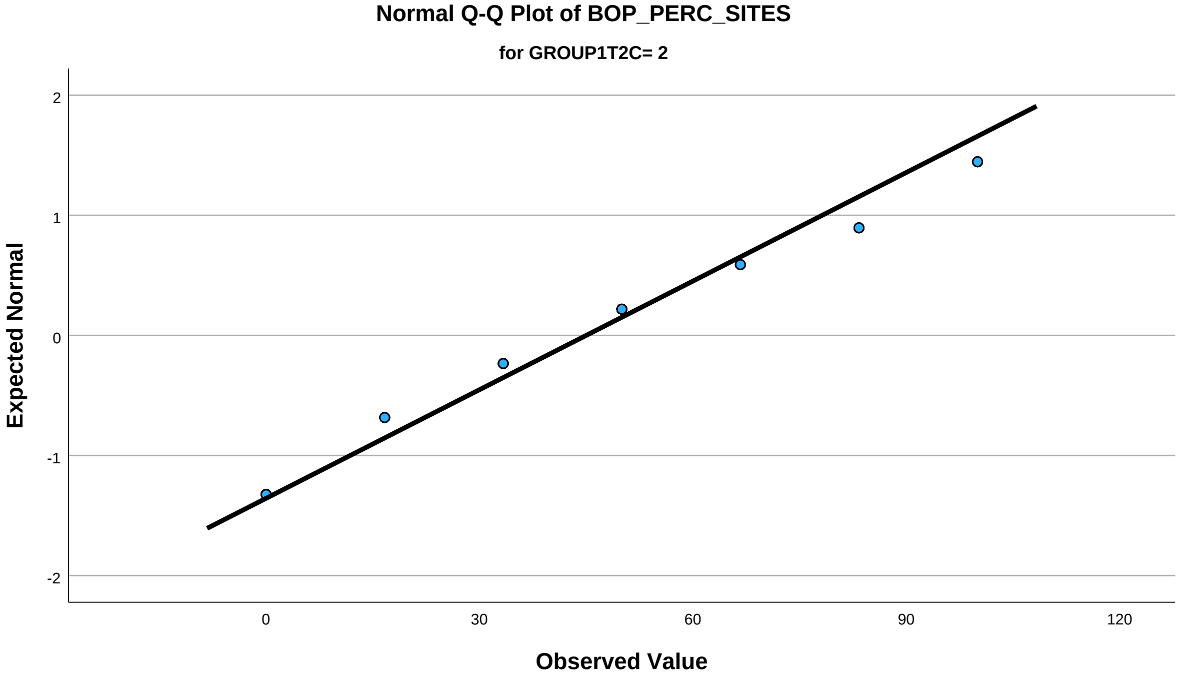

Detrended Normal Q-Q Plots

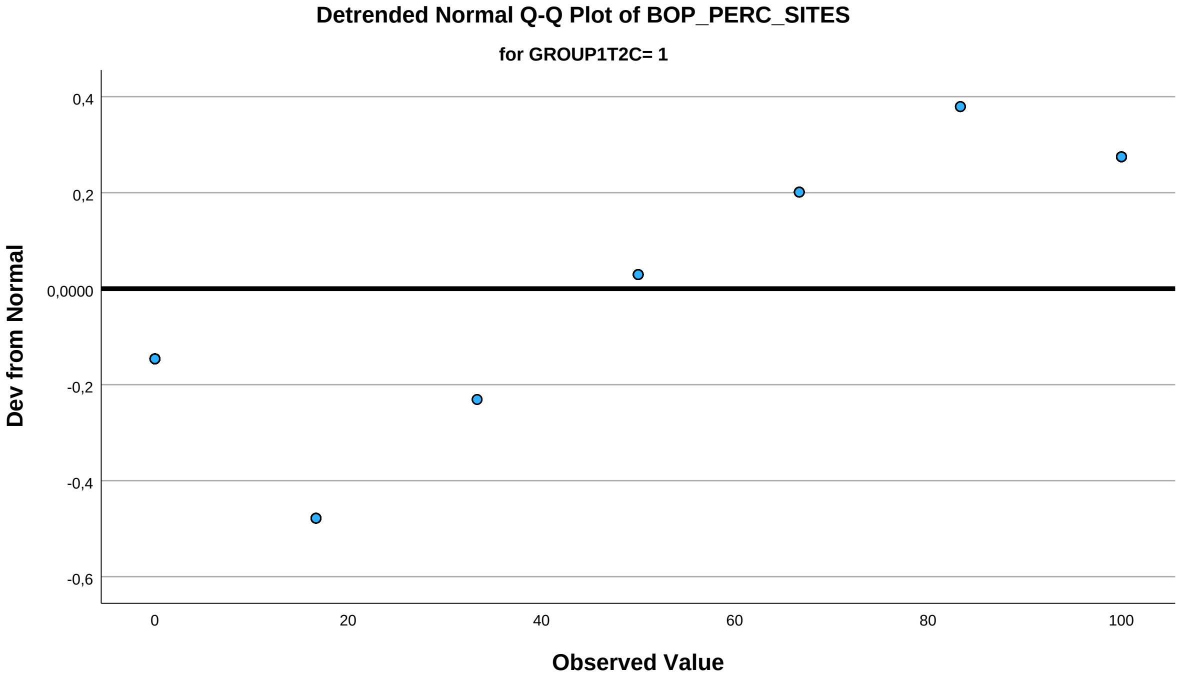
Detrended Normal Q-Q Plot of BOP_PERC_SITES
for GROUP1T2C= 1
Dev from Normal
0,4
0,2
0,0000
-0,2
-0,4
-0,6
0
20
40
60
80
100
Observed Value

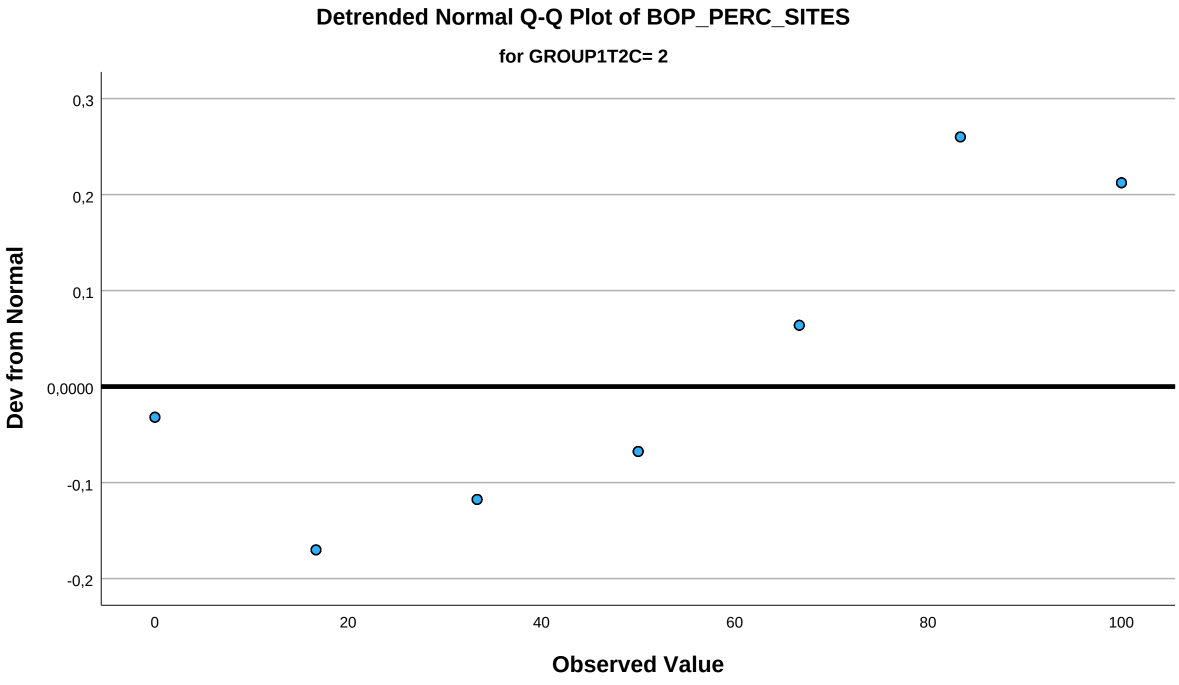
Detrended Normal Q-Q Plot of BOP_PERC_SITES
for GROUP1T2C= 2
Dev from Normal
0,3
0,2
0,1
0,0000
-0,1
-0,2
0
20
40
60
80
100
Observed Value

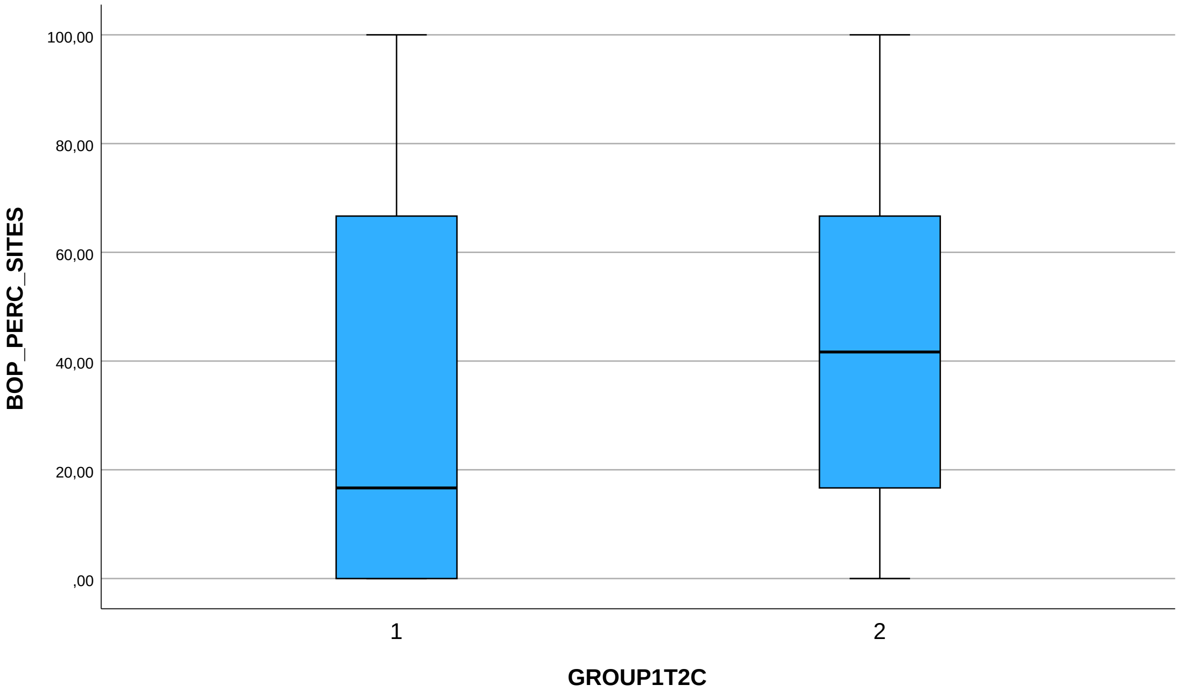
100,00
80,00
60,00
40,00
20,00
,00
BOP_PERC_SITES
1
2
GROUP1T2C
